# Proton pencil beam scanning reduces secondary cancer risk in breast cancer patients with internal mammary chain involvement compared to photon radiotherapy

**DOI:** 10.1186/s13014-020-01671-8

**Published:** 2020-10-02

**Authors:** Giorgio Cartechini, Francesco Fracchiolla, Loris Menegotti, Emanuele Scifoni, Chiara La Tessa, Marco Schwarz, Paolo Farace, Francesco Tommasino

**Affiliations:** 1 Department of Physics, University of Trento, Via Sommarive, 14, 38123 Povo, TN Italy; 2 Trento Institute for Fundamental Physics and Applications (TIFPA), National Institute for Nuclear Physics, (INFN), Povo, Italy; 3 Protontherapy Department, Azienda Provinciale per i Servizi Sanitari (APSS), Trento, Italy; 4 Health Physics Department, Azienda Provinciale per i Servizi Sanitari (APSS), Trento, Italy

**Keywords:** Secondary cancer risk, Breast cancer, Proton therapy, Tangential 3D-CRT, VMAT

## Abstract

**Purpose:**

Proton pencil beam scanning (PBS) represents an interesting option for the treatment of breast cancer (BC) patients with nodal involvement. Here we compare tangential 3D-CRT and VMAT to PBS proton therapy (PT) in terms of secondary cancer risk (SCR) for the lungs and for contralateral breast.

**Methods:**

Five BC patients including supraclavicular (SVC) nodes in the target (Group 1) and five including SVC plus internal-mammary-nodes (IMNs, Group 2) were considered. The Group 1 patients were planned by PT versus tangential 3D-CRT in free-breathing (FB). The Group 2 patients were planned by PT versus VMAT considering both FB and deep-inspiration breath hold (DIBH) irradiation. The prescription dose to the target volume was 50 Gy (2 Gy/fraction). A constant RBE = 1.1 was assumed for PT. The SCR was evaluated with the excess absolute risk (EAR) formalism, considering also the age dependence. A cumulative EAR was finally computed.

**Results:**

According to the linear, linear-exponential and linear-plateau dose response model, the cumulative EAR for Group 1 patients after PT was equal to 45 ± 10, 17 ± 3 and 15 ± 3, respectively. The corresponding relative increase for tangential 3D-CRT was equal to a factor 2.1 ± 0.5, 2.1 ± 0.4 and 2.3 ± 0.4. Group 2 patients showed a cumulative EAR after PT in FB equal to 65 ± 3, 21 ± 1 and 20 ± 1, according to the different models; the relative risk obtained with VMAT increased by a factor 3.5 ± 0.2, 5.2 ± 0.3 and 5.1 ± 0.3. Similar values emerge from DIBH plans. Contrary to photon radiotherapy, PT appears to be not sensitive to the age dependence due to the very low delivered dose.

**Conclusions:**

PBS PT is associated to significant SCR reduction in BC patients compared to photon radiotherapy. The benefits are maximized for young patients with both SVC and IMNs involvement. When combined with the improved sparing of the heart, this might contribute to the establishment of effective patient-selection criteria for proton BC treatments.

## Introduction

The possibility to exploit proton therapy (PT) for the treatment of breast cancer (BC) patients has received growing interest in recent years. This is demonstrated also by the recent results of the first prospective clinical trial comparing photon versus proton radiation therapy for the treatment of BC with nodal involvement [1]. Considering that photon radiotherapy provides good results in terms of local control and 5-year survival in early-stage BC patients [2], the interest toward the use of protons is motivated mainly by the possibility to significantly spare organs at risk (OARs) distal to the tumour, namely the heart and the lungs, especially in left-side BC patients with nodal involvement for which higher doses are expected [3]. In fact, cardiac toxicity represents historically a concern for BC radiotherapy. The evidence of an increased risk for radiation-induced heart diseases previously reported by Darby et al. [4] has been confirmed by the recent analysis published by Taylor et al. [5].

In terms of distal OAR sparing, the dosimetric advantages offered by protons are obvious [6]. However, the non-linear dependence between dosimetric parameters and toxicity outcomes has raised the need to demonstrate that the OARs sparing offered by protons actually translates into a reduced rate of expected toxicity. Several in silico studies applied different modelling approaches in order to quantify the potential gain in terms of reduced normal tissue complication probability (NTCP) when PT is used for BC treatment [7–10]. Overall, these works showed that a significant NTCP reduction is expected for lung and heart toxicity, with a benefit more or less pronounced depending on the specific endpoint considered. Nowadays, these types of analysis are considered of central importance for establishing an effective patient allocation according to the so-called model-based selection criteria [11, 12].

In view of the long life expectancy that modern treatments can offer to early-stage BC patients, the question has been recently posed whether radiation-induced secondary cancer risk (SCR) could be a concern, especially for young patients [13–15]. While extensive efforts have been devoted to minimize the risks of heart toxicity (e.g. the introduction of deep inspiration breath hold [DIBH] techniques [16]), the same attention level was not directed toward SCR reduction until recently. Remarkably, the study by Hoekstra et al. [17] indicates that whole-breast radiotherapy could translate into a 2.9% excess mortality due to secondary lung cancer, which is higher than the expected mortality due to late heart toxicity. While in the past a comparably high SCR was attributed to the contralateral breast [18], the data presented in 2014 by Abo-Madyan et al. indicate that with current treatment techniques the main source of SCR after radiotherapy in BC patients is the ipsilateral lung, and that higher risk is expected after radiotherapy with photons when the VMAT technique is used compared to tangential 3D-CRT [19]. Despite the low absolute incidence of SCR in BC treatments, these studies suggest that such risk could be not negligible, and that mitigation strategies might consists in the selection of the most suitable radiotherapy technique for each patient, accounting for both anatomical and clinical characteristics. In this regard, one main issue is the nodal involvement and particularly the inclusion in the target of internal mammary nodes (IMNs). In fact, the requirement to irradiate the IMNs increases the exposure of nearby critical organs, especially the heart and the ipsilateral lung. For such patients, VMAT has been shown to be effective in reducing dose to the heart and lungs while generating more conformal isodose distributions with respect to tangential photon techniques [20].

In the present study, we investigate how pencil beam scanning PT can reduce the SCR in BC patients with different degree of nodal involvement treated with radiotherapy. Two photon techniques, namely 3D-CRT and VMAT, as well as pencil beam scanning (PBS) PT were employed. The dose distributions were converted into an excess absolute risk (EAR) for ipsilateral and contralateral lung, as well as for contralateral breast. Based on both the cumulative and organ-level EAR, the different irradiation techniques were finally compared in terms of SCR induction.

## Methods

### Patients and treatment planning

Ten left side BC patients with nodal involvement previously treated with photons (3D-CRT/VMAT) at our institution were included in the study and then separated in two different groups for the purpose of our analysis. Five of these patients including the nodes in the target were assigned to Group 1, while the other five including both the SVC and IMNs were allocated to Group 2. For the first group PT was compared to tangential 3D-CRT, while VMAT was employed as photon technique for the latter, due to the more complex target geometry [20]. The average patient age at the time of treatment was 57 ± 8 and 54 ± 11 for Group 1 and 2, respectively. Three patients in Group 1 and two in Group 2 had breast implant after previous surgery, while one patient in Group 2 was treated post-mastectomy.

PT treatment plans were calculated and optimized with RayStation TPS (version 6.0.0.24, RaySearch Laboratories AB, Stockholm, Sweden), assuming a constant RBE of 1.1 and using a Monte Carlo (MC) dose-engine to compute the dose distribution (associated statistical uncertainty set to 1%). Beam energies in the range 70–228 meV are available at our institution. Assuming a Gaussian spot size, beam full width half maximum (FWHM) in air at isocenter varies from about 16 mm to about 4 mm at the lowest and highest energies, respectively (for more information see also [21]). All plans consisted in a single 30° beam, including the use of a range shifter (4.08 cm water-equivalent thickness) mounted on a movable snout. The beam arrangement and the optimization criteria for target volume and OARs were adopted from the previous work by Depauw et al. [22]. Preliminary work included the verification of the resulting dose distributions and the estimation of neutron dose contribution with an independent MC tool based on TOPAS previously validated at our institution [21].

Photon treatment plans with tangential 3D-CRT and VMAT techniques were calculated and optimized with TPS Monaco (version 5.11.02 Elekta, Sweden). A MC dose-engine was used to compute the VMAT dose distribution (associated statistical uncertainty 1%), while a Collapsed Cone Convolution (CCC) algorithm was employed for tangential 3D-CRT. For the VMAT technique, we used two partial-arcs (about 240°), while the tangential 3D-CRT plans were based on field-in-field tangential technique [23] plus an anterior field for SVC lymph nodes.

A dose grid of 2 mm^3^/voxel was adopted for all plans. The same prescription dose of 50 Gy in 2 Gy/fraction was assumed for each treatment technique. Based on conventional CT imaging, treatment plans were optimized either in free breathing (FB) or with DIBH (the latter for Group 2 only).

For simplicity, all doses are expressed in Gy. For protons, these are Gy(RBE) values as they include the multiplication by the constant RBE factor.

### Risk modelling

Our interest was focused on the calculation of SCR for ipsilateral lung and for contralateral lung and breast. The excess absolute risk (EAR) formalism proposed by Schneider was adopted to quantify the SCR associated to each plan [24, 25]. This is based on combining a baseline risk at low doses (EAR_0_) derived from A-bomb survivor data with the concept of Organ Equivalent Dose (OED), assuming a direct proportionality:

1 $$EAR = EAR_{0} \cdot OED$$

The EAR for single OARs was computed considering exposure at two different ages, namely at 30 and 50 years, while keeping fix the attained age of 70 years. This can be done by a proper selection of the EAR_0_ value. According to the analysis of the A-bomb survivors presented by Preston et al. [26], the EAR_0_ for lung and breast at low doses in females is equal to 7.5 (95% CI 5.1–10.0) and 9.2 (95% CI 6.8–12.0) cases per 10,000 persons per year per Gy at the age of 70 years, assuming exposure at the age of 30 years; these values are modified into 7.8 (95% CI 4.6–12) for the lung and 3.7 (95% CI 2.1–5.9) for the breast for exposure at the age of 50 years. A cumulative EAR was also computed, corresponding to the sum of EAR for single organs and thus reflecting also age dependence.

The concept of OED assumes that all dose distributions in an organ are equivalent if they cause the same radiation-induced cancer incidence [24]. The OED is thus calculated combining the information contained in the differential DVHs with a dose–response curve describing the radiation-induced cancer induction. Three different dose–response models were adopted in this study, namely the linear, linear-exponential and linear-plateau models [27, 28]:

2 $$OED_{linear} = \frac{1}{{V_{T} }}\sum DVH\left( {D_{i} } \right) \cdot D_{i}$$

3 $$OED_{linear - exp} = \frac{1}{{V_{T} }}\sum DVH\left( {D_{i} } \right) \cdot D_{i} \cdot e^{{ - \alpha D_{i} }}$$

4 $$OED_{linear - plateau} = \frac{1}{{V_{T} }}\sum DVH\left( {D_{i} } \right) \cdot \left( {1 - e^{{ - \delta D_{i} }} } \right)/\delta$$

where α = 0.044 Gy^−1^, δ = 0.139 Gy^−1^ are parameters estimated by a combined fit to A-bomb and Hodgkin lymphoma survivors [29]. The three different models adopted to describe OED dependence on dose correspond to different assumptions on the underlying dose response curve. Specifically, the linear model assumes a simple linear dependence of OED on dose. On the contrary, the linear-exponential and linear-plateau models take into account the possibility of the cell to repair/repopulate after irradiation. More in detail, the two approaches correspond to the extreme cases: no repair/repopulation is assumed by the linear-exponential model, while full repair/repopulation is assumed by the linear-plateau models [27, 28]. Even though associated to an overestimation of the SCR for OAR doses above few Gy, and therefore considered out-dated by some authors [30, 31], the EAR values obtained with the linear model are also reported for reference.

### Statistical analysis

The statistical difference between the EARs obtained with the different treatment techniques was investigated. Specifically, two-tail paired Student *t* tests were performed comparing the EARs associated to the treatment techniques two by two. The difference was considered significant when *p* < 0.05.

## Results

**Fig. 1:**
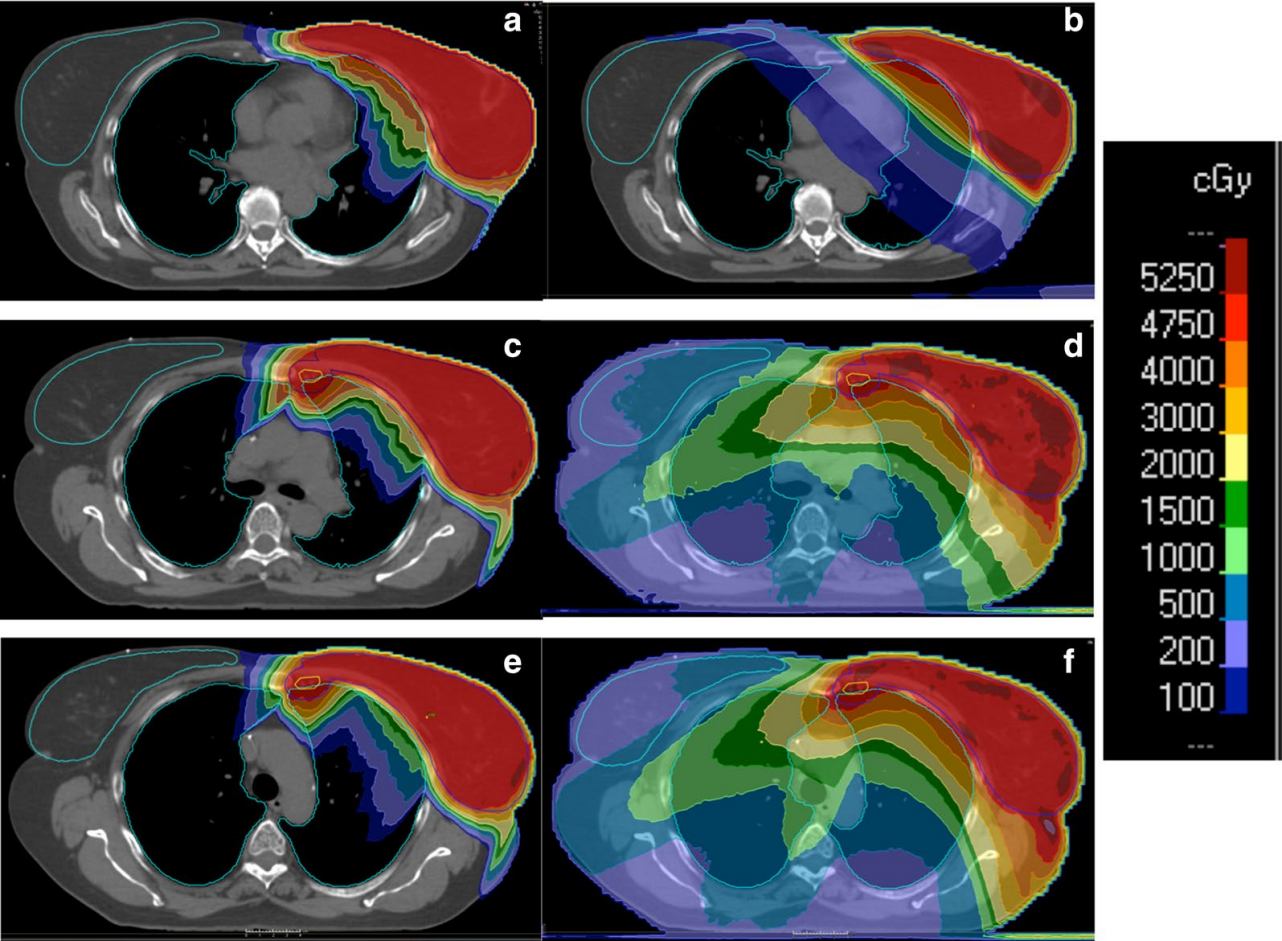
Summary of the dose distributions obtained with the different irradiation techniques. A representative Group 1 patient receiving PT is shown in **a**, while the corresponding 3D-CRT plan is displayed in **b**. Concerning Group 2, a typical dose distribution with FB treatment is shown for PT (**c**) and VMAT (**d**). The corresponding DIBH plan is finally reported for PT (**e**) and VMAT (**f**). The limited dependence on patient setup (FB vs DIBH) can be appreciated

**Fig. 2:**
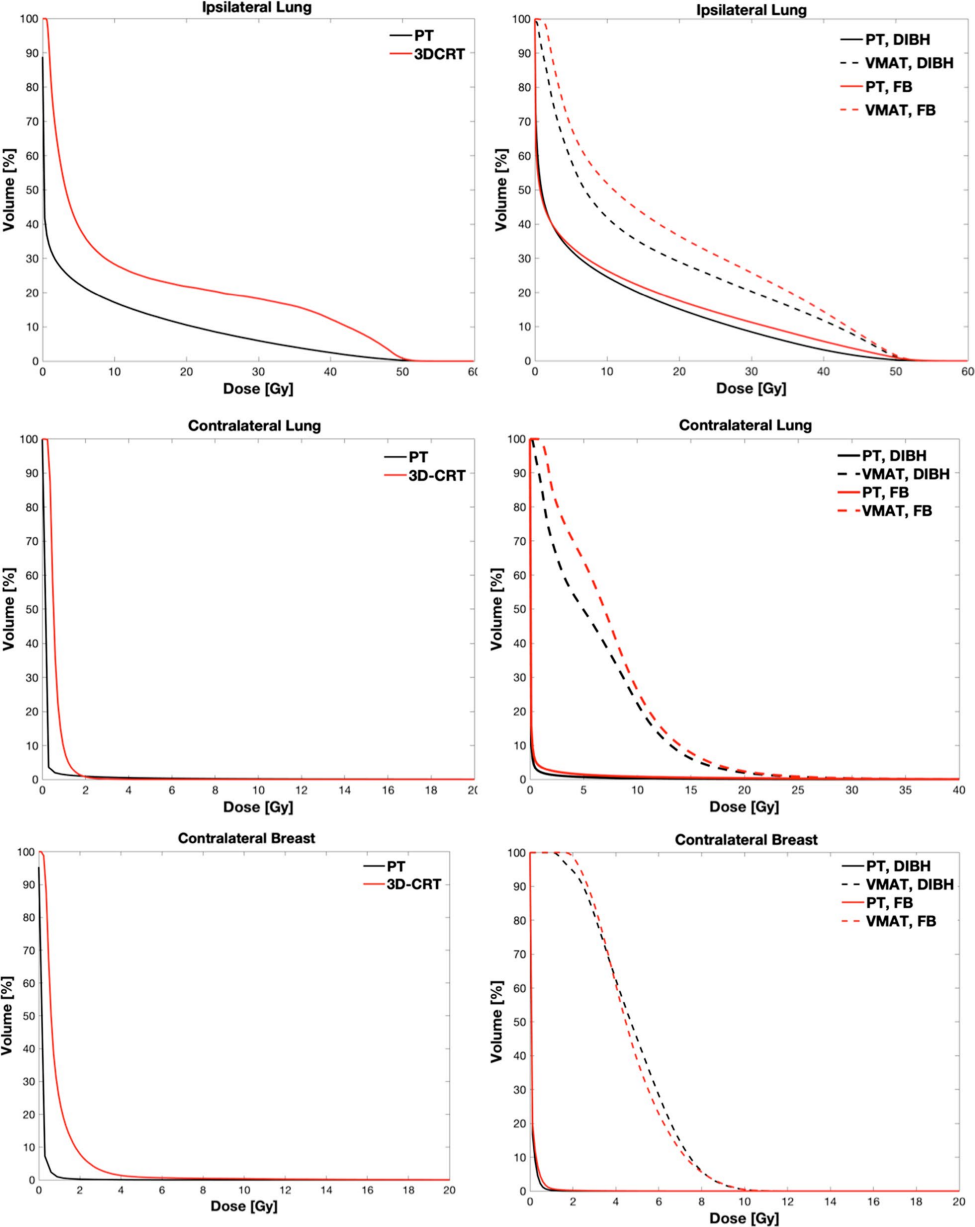
Cumulative DVHs obtained averaging over the patients included in the study for Group 1 (left column) and Group 2 (right column). DVHs are shown for the ipsilateral lung (upper panel), contralateral lung (middle panel) and contralateral breast (lower panel). The different planning techniques are shown for each OAR. Please notice that different scales are adopted for the X-axis

The dose distributions obtained with the different irradiation modalities are illustrated in Fig. 1 for a representative patient. The results indicate that a comparable and good coverage of the target volume is obtained independently of the radiotherapy technique or patient setup. The better conformity of PT is evident, as well as the significantly lower dose bath out-of-field compared to 3D-CRT and even more to VMAT. The OAR doses obtained with the different techniques are summarized in Table 1. As expected, the data indicate that OARs receive overall lower dose for Group 1, when SVC nodes only are included in the target. In this case, tangential 3D-CRT delivers a dose about 3 times higher than PT to the ipsilateral lung, while comparable doses below 1 Gy are obtained for the contralateral organs. Compared to Group 1, the PT dose to the ipsilateral lung increases by a factor 2.5–2.8 in Group 2 due to IMNs involvement, with a low dependence on patient setup (either FB or DIBH irradiation). Concerning photon irradiation, a dose about twice as high is observed for VMAT compared to PT. Remarkable differences between PT and VMAT are observed for contralateral OARs: while an average dose well below 1 Gy is delivered by PT, VMAT results in average doses of about 6–7 Gy and 5 Gy for the contralateral lung and breast, respectively, with a minor dependence on FB or DIBH. The dosimetric outcomes are further evidenced by the average cumulative DVHs reported in Fig. 2 for both treatment groups (the corresponding differential DVHs are reported in Additional file 1: Supplementary Figure S1).

**Table 1:** Dosimetric parameters for the OARs included in the SCR estimation for the patients included in the analysis

	FB				DIBH	
	Group 1		Group 2		Group 2	
	PT	Tangential 3D-CRT	PT	VMAT	PT	VMAT
Average dose (Gy)						
Ipsilateral lung	5.9 ± 2.1	11.5 ± 1.1	8.4 ± 0.3	16.4 ± 1.6	7.4 ± 0.8	14.6 ± 0.9
Contralateral lung	0.08 ± 0.10	0.6 ± 0.1	0.3 ± 0.1	7.3 ± 0.4	0.15 ± 0.02	6.2 ± 0.3
Contralateral breast	0.08 ± 0.03	0.9 ± 0.1	0.09 ± 0.02	4.7 ± 0.3	0.07 ± 0.01	4.8 ± 0.3

The data are presented as mean ± standard error of the mean (SEM, n = 5)

The properties of the dosimetric data reflect into the predicted EAR for the three OARs considered. EAR_30–70_ relative to the FB irradiation with PT and tangential 3D-CRT for Group 1 are shown in Table 2 (Additional files 1, 2, 3: Supplementary Figures S1–S4 show both average and single-patient EAR variation for Group 1 and Group 2, the latter with FB and with DIBH, respectively). Independently of the dose response model adopted for the EAR calculation, we always observe the same trend of a lower SCR for PT compared to tangential 3D-CRT. When paired significance tests were performed, a significant difference was always observed between the techniques. For PT, the largest EAR component originates from the ipsilateral lung, with the SCR getting close to zero for the contralateral OARs due to the very low doses. For tangential 3D-CRT, a non-negligible fraction of EAR is due to the dose deposited in the contralateral lung and breast, in the order of about 10% for the linear model and of about 30% for linear-shape and plateau models.

**Table 2:** Average EAR expressed in number of cases per 10,000 persons per year for Group 1 and Group 2

	EAR_30-70_ (number of cases per 10,000 persons per year)					
	3D-CRT			PT		
	Linear	Lin-exp	Lin-plat	Linear	Lin-exp	Lin-plat
Group 1						
Ipsilateral lung	85 ± 18	27 ± 3	25 ± 3	44 ± 22	16 ± 7	14 ± 6
Contralateral lung	3 ± 1	3 ± 1	3 ± 1	1 ± 1	0 ± 1	0 ± 1
Contralateral breast	7 ± 3	6 ± 2	6 ± 2	0 ± 0	0 ± 0	0 ± 0

	VMAT			PT		
	Linear	Lin-exp	Lin-plat	Linear	Lin-exp	Lin-plat
Group 2—FB						
Ipsilateral lung	131 ± 17	40 ± 3	37 ± 3	63 ± 5	20 ± 3	18 ± 2
Contralateral lung	55 ± 8	35 ± 3	30 ± 2	2 ± 2	1 ± 1	1 ± 1
Contralateral breast	44 ± 6	35 ± 4	31 ± 3	0 ± 0	0 ± 0	0 ± 0
Group 2—DIBH						
Ipsilateral lung	108 ± 15	35 ± 3	33 ± 3	53 ± 11	20 ± 2	17 ± 2
Contralateral lung	46 ± 4	29 ± 2	25 ± 1	1 ± 0	1 ± 0	1 ± 0
Contralateral breast	43 ± 7	34 ± 5	31 ± 4	0 ± 0	0 ± 0	0 ± 0

EAR are reported for the three OARs considered, according to the linear, linear-exponential and linear-plateau models. Uncertainty indicates the standard deviation

The EAR values obtained for Group 2 are reported in Table 2 for both FB and DIBH irradiation. First, when considering the EAR associated to PT in FB, we observe an increased risk compared to Table 2. This is a consequence of the more complex target geometry, resulting into higher delivered doses (see also Table 1). In detail, for PT the EAR with IMNs inclusion increases by a about 28% according to the linear-exponential and plateau models (about 43% increase with the linear model). The increased risk is largely due to the ipsilateral lung component, while contralateral OARs are less affected. Table 2 also shows that the risk associated to VMAT irradiation is about 5 times higher compared to PT according to the linear-exponential and plateau models (about 3.5 higher for the linear model). *p* values lower than 0.01 were obtained for all the paired comparisons. Contrary to PT irradiation, with VMAT each OAR contributes for about 1/3 to the overall EAR, as a consequence of the extended distribution of low- and intermediate-doses. The impact of target complexity is also evident when comparing the SCR for tangential 3D-CRT and VMAT with FB irradiation, resulting in an EAR about 3 times larger for the latter. Finally, no large differences emerge in Group 2 patients when DIBH is employed, which results in about 10% EAR decrease compared to FB.

**Fig. 3:**
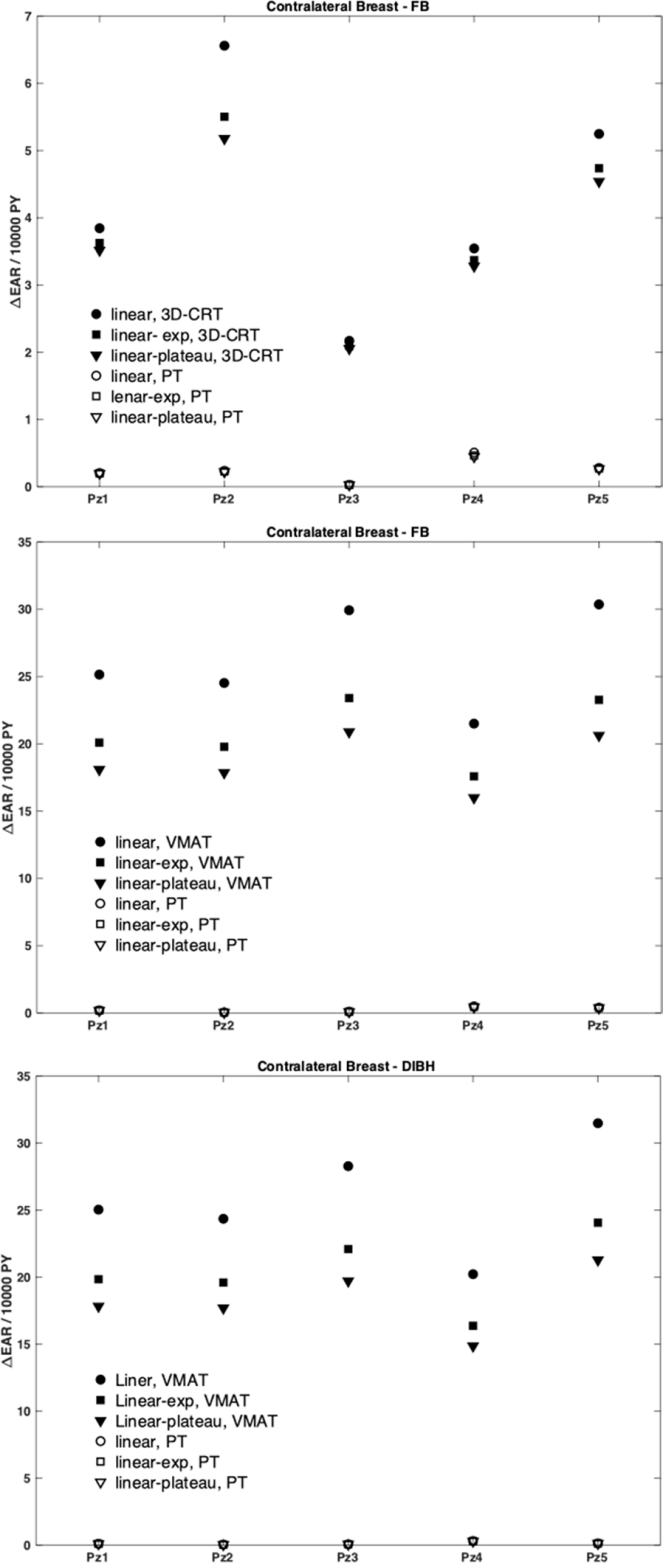
EAR difference as a function of the age of exposure (EAR_30–70_–EAR_50–70_) for the single patients included in the analysis. The EAR difference is shown for the contralateral breast for Group 1 patients treated with tangential 3D-CRT vs PT (top panel) and for Group 2 patients treated with VMAT versus PT in the case of FB (middle panel) and DIBH (bottom panel)

While the corresponding EAR_50–70_ can be obtained by simply scaling the EAR_0_ coefficients (see “Risk modelling” section), it is interesting to evaluate the difference in EAR obtained for the same patient, assuming the irradiation at an age of 30 or 50 years, while keeping the attained age of 70 years constant. Delta-EAR (ΔEAR = EAR_30–70 _− EAR_50–70_) was thus computed and the data are summarized in Fig. 3.

The ΔEAR associated to the contralateral breast is remarkably higher for Group 2 patients treated with VMAT, with absolute values depending on the dose–response model and minor difference between FB and DIBH. This is a consequence of the marked EAR_0_ reduction for older patients (9.2 vs 3.7 cases per 10,000 persons per year per Gy). In details, data show a reduction in contralateral breast SCR for VMAT by about 60% for patients exposed at 50 rather than at 30 years. A smaller effect is observed for tangential 3D-CRT in Group 1, which is explained by the lower OAR doses due to the different target, while no substantial changes are always associated to PT, independently on the specific target configuration, because of the very low dose released to the contralateral breast and the extremely low EAR associated. Due to minor difference in EAR_0_ (7.5 vs 7.8 cases per 10,000 persons per year per Gy), a minor ΔEAR is attributed to the lungs (not shown).

## Discussion

The increasing attention towards radiation induced SCR in BC patients with nodal involvement has highlighted the attractive potential of pencil beam scanning PT in minimizing the low-dose bath compared to photon radiotherapy. In this scenario, we performed a systematic analysis to compare the expected EAR for such patients. PT was compared to tangential 3D-CRT for treatment including SVC nodes (Group 1), and to VMAT when both SVC and IMNs nodes were involved (Group 2). For the latter, it was previously shown that VMAT is the preferred technique compared to tangential 3D-CRT, which in order to provide acceptable target coverage would result into unacceptable high doses to the ipsilateral lung [6, 20, 32].

Different dose–response models were tested on three OARs, including also a partial study of the age dependence. Overall our results confirm the expectation of a significant reduction of SCR after treatment with PT compared to both tangential 3D-CRT and VMAT, with specific differences that we are going to discuss.

In terms of cumulative EAR_30–70_ per 10,000 patients for Group 1, PT resulted in the expected incidence (average value ± SEM) of 45 ± 10, 17 ± 3 and 15 ± 3 according to the linear, linear-exponential and linear-plateau model, respectively. Compared to PT, the corresponding relative increase in the expected incidence for tangential 3D-CRT was by a factor 2.1 ± 0.5, 2.1 ± 0.4 and 2.3 ± 0.4. However, when excluding the linear model that likely overestimates the risk, we notice that absolute risk values are comparably low for Group 1. The two-fold risk increase due to tangential 3D-CRT is therefore associated to a limited impact on SCR. Moreover, this risk could be further reduced by the use of DIBH for tangential 3D-CRT irradiation, which in addition to lower heart dose also results in a reduced ipsilateral lung dose [6, 33–35].

Due to the enhanced target complexity, for Group 2 patients the EAR_30-70_ values with PT increase to 65 ± 3, 21 ± 1 and 20 ± 1 according to the linear, linear-exponential and plateau model, respectively. The need to preserve sufficient target coverage when such patients are treated with VMAT leads to a dose bath that is responsible for a corresponding risk increase by a factor 3.5 ± 0.2, 5.2 ± 0.3 and 5.1 ± 0.3 compared to PT. A minor dependence is registered on FB versus DIBH patient setup, with the tendency of a slight risk reduction for the latter. Specifically, when excluding the linear risk model, very similar EARs are obtained from the comparison of Group 2 PT plans with and without DIBH. This indicates that, while DIBH improves OAR sparing in VMAT plans, the increased treatment complexity might not be justified for PT, where no significant benefit is expected in terms of SCR. The opportunities offered by VMAT in the treatment of BC are now established, with several studies demonstrated that VMAT is the optimal photon technique in terms of target coverage, especially when IMNs are included [6, 20, 32]. In a study dedicated to SCR evaluation with different photon techniques in the presence of setup uncertainties, Zhang et al. [36] show that the improved target coverage of VMAT comes a the price of an increased SCR. At the same time, VMAT is also associated to lower heart doses compared to tangential 3D-CRT, especially when combining with DIBH [6]. However, taken together with the information emerging from the age-dependence analysis, our data indicate that the gain in terms of lower SCR with PT is maximized for younger patients with IMNs involvement. Combined with the reduced mean heart dose, this could support an efficient patient selection for PT treatments.

A limited amount of published data is currently available for comparison. The data presented here for photons are consistent with the previous work on photon irradiation published by Abo-Madyan et al. [19], where similar relative risks were obtained when comparing VMAT to tangential 3D-CRT, while the absolute incidence turned out to be higher in our study due to the inclusion of lymph nodes in the target volume. Concerning PT, De Rose et al. published some data as a part of a treatment planning study. When comparing pencil beam scanning PT to VMAT for BC patients with nodal involvement treated in FB, similar results were obtained, with slight differences in the absolute values that are also due by the different selection of model parameters [37]. Recently Paganetti et al. [38] presented an analysis dedicated to SCR in BC patients receiving the three radiation modalities that we also investigated in this study. Due to the different selection of model parameters and patient characteristics, the data can be only partially compared. However, the data clearly show that the expected SCR is highest for VMAT and for the ipsilateral lung. In addition to the lungs and breast, a low EAR was associated also with the oesophagus and thyroid. This risk was found to be one or more order of magnitudes lower compared to the ipsilateral lung EAR. Compared to the work by Paganetti et al., by means of the separation into Group 1 and 2 based on target complexity, the present study allows identifying the weight of nodal involvement on the SCR reduction offered by protons, showing that the benefit is maximized when both SVC and IMNs belong to the target.

Some approximations have been assumed in this study. First of all, the EAR estimation is affected by comparably large uncertainties, which are historically due to the difficult determination of the EAR_0_ coefficients and to their application to a specific patients’ cohort. This is known to be an issue in the SCR estimation but it does not affect the relative comparison among different techniques, which is the main target of the present analysis. The EAR evaluations performed in this study are based on three different dose–response models (see “Methods” section). The application of the linear model resulted always in higher EAR compared to the bell-shape or plateau models. However, the linear model is expected to fail at doses higher than a few Gy, resulting in an overestimation of the SCR [30].

The neutron contribution to the overall dose was not included in the analysis. Previous studies indicate that a negligible increase due to neutron dose in photon BC treatments [39]. Concerning the use of protons, it was shown that the use of pencil beam scanning PT is associated to a significant reduction in the production of secondary neutrons compared to the passive scattering techniques. Independent MC simulations based on the TOPAS toolkit [40] were performed in preparation of this works, and indicated that a neutron dose on the order of 1 mSv/treatment Gy was associated to the OARs, in agreement with literature [41]. Thus, overall neutron doses would have a minor impact on EAR estimation. The impact of CT imaging on SCR was also neglected in this study.

Finally, variable RBE was not included in this study. Indeed the impact of variable RBE might be two-fold: on the one hand it could lead to an increased mutation rate [42], while on the other hand it would translate into an increased biological dose. While the former aspect can be associated to an expected increase in SCR, the latter will also increase the probability of cell inactivation, which in turn decreases the SCR [43]. The effect of variable RBE on SCR is not explicitly taken into account in the current EAR models and would deserve a dedicated investigation. At the same time, the recent study published by Raptis et al. [44], even though based on a different modelling, indicates that the inclusion of a variable RBE does not lead to a considerable SCR increase. This suggests that for BC patients RBE might have a second-order effect for the latter specific endpoint.

## Conclusions

Our work indicates a benefit from the use of PT in BC patients with nodal involvement. When coupled with the lower NTCP expected for distal OARs (i.e. heart, lung), this represents valuable information for the establishment of cost-effective patient selection criteria for BC treatment. Specifically, we show that the gain offered by PT is maximized when the target volume includes IMNs. In this setting and especially for younger patients, PT might be an alternative to VMAT irradiation.

## Supplementary information

**Additional file 1: Figure S1.** Differential DVHs obtained averaging over the patients included in the study for Group 1 (left column) and Group 2 (right column). DVHs are shown for the ipsilateral lung (upper panel), contralateral lung (middle panel) and contralateral breast (lower panel). The different planning techniques are shown for each OAR. Please notice that different scales are adopted for the X-axis.

**Additional file 2: Figure S2.** Average (black squares) and single-patient (blue lines) EAR calculated for the three OARs included in the analysis for tangential 3D-CRT vs PT. Data refer to plans calculated in FB for Group 1. The data displayed refer to patients receiving radiotherapy at the age of 30 years and attaining the age of 70 years. Error bars indicate standard deviation. According to the different absolute values, the Y-axis scale changes for the different OARs.

**Additional file 3: Figure S3.** Average (black squares) and single-patient (blue lines) EAR calculated for the three OARs included in the analysis for VMAT vs PT. Data refer to plans calculated in FB for Group 2. The data displayed refer to patients receiving radiotherapy at the age of 30 years and attaining the age of 70 years. Error bars indicate standard deviation. According to the different absolute values, the Y-axis scale changes for the different OARs.

**Additional file 4: Figure S4.** Average (black squares) and single-patient (blue lines) EAR calculated for the three OARs included in the analysis for VMAT vs PT. Data refer to plans calculated with DIBH for Group 2. The data displayed refer to patients receiving radiotherapy at the age of 30 years and attaining the age of 70 years. Error bars indicate standard deviation. According to the different absolute values, the Y-axis scale changes for the different OARs.

## Data Availability

Please contact author for data requests.
