# Supplementary figures and images for: Proton pencil beam scanning reduces secondary cancer risk in breast cancer patients with internal mammary chain involvement compared to photon radiotherapy
Source: Radiat Oncol. 2020 Oct 2;15:228. doi: 10.1186/s13014-020-01671-8 (PMC7532613; doi:10.1186/s13014-020-01671-8)

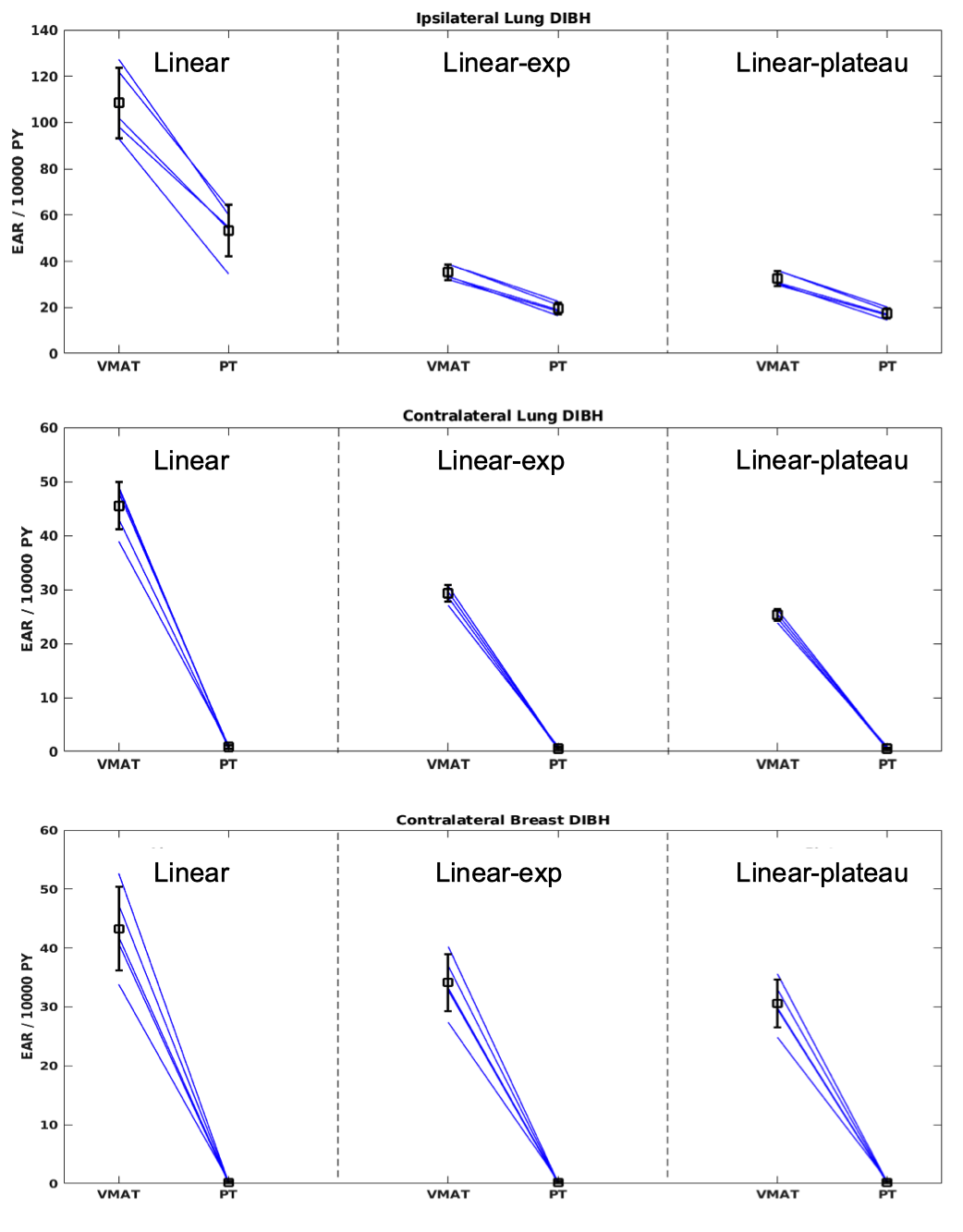

Supplement: Supplementary file 1 — Additional file 1: Figure S1. Differential DVHs obtained averaging over the patients included in the study for Group 1 (left column) and Group 2 (right column). DVHs are shown for the ipsilateral lung (upper panel), contralateral lung (middle panel) and contralateral breast (lower panel). The different planning techniques are shown for each OAR. Please notice that different scales are adopted for the X-axis. [file 13014_2020_1671_MOESM1_ESM.png]

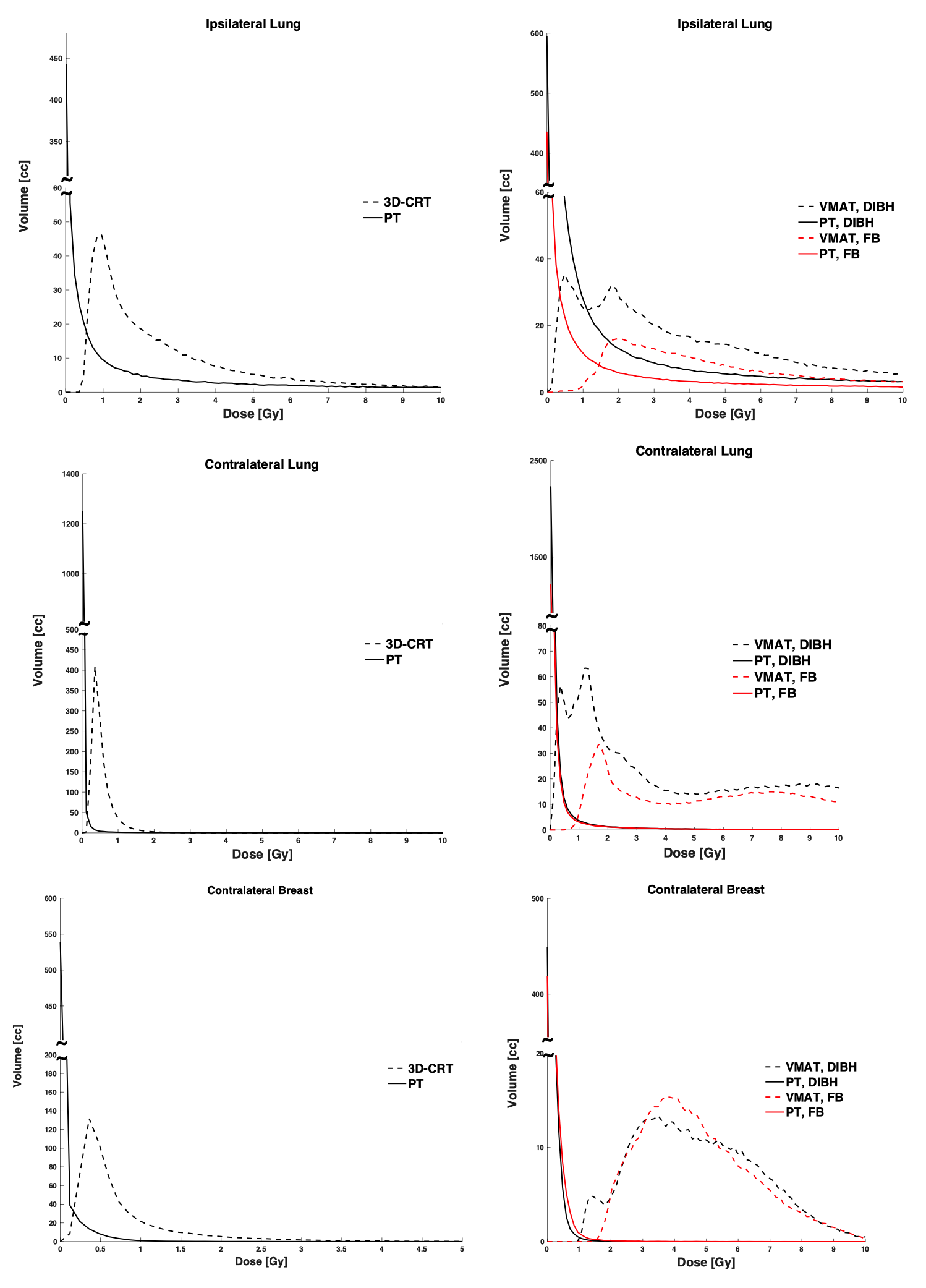

Supplement: Supplementary file 2 — Additional file 2: Figure S2. Average (black squares) and single-patient (blue lines) EAR calculated for the three OARs included in the analysis for tangential 3D-CRT vs PT. Data refer to plans calculated in FB for Group 1. The data displayed refer to patients receiving radiotherapy at the age of 30 years and attaining the age of 70 years. Error bars indicate standard deviation. According to the different absolute values, the Y-axis scale changes for the different OARs. [file 13014_2020_1671_MOESM2_ESM.png]

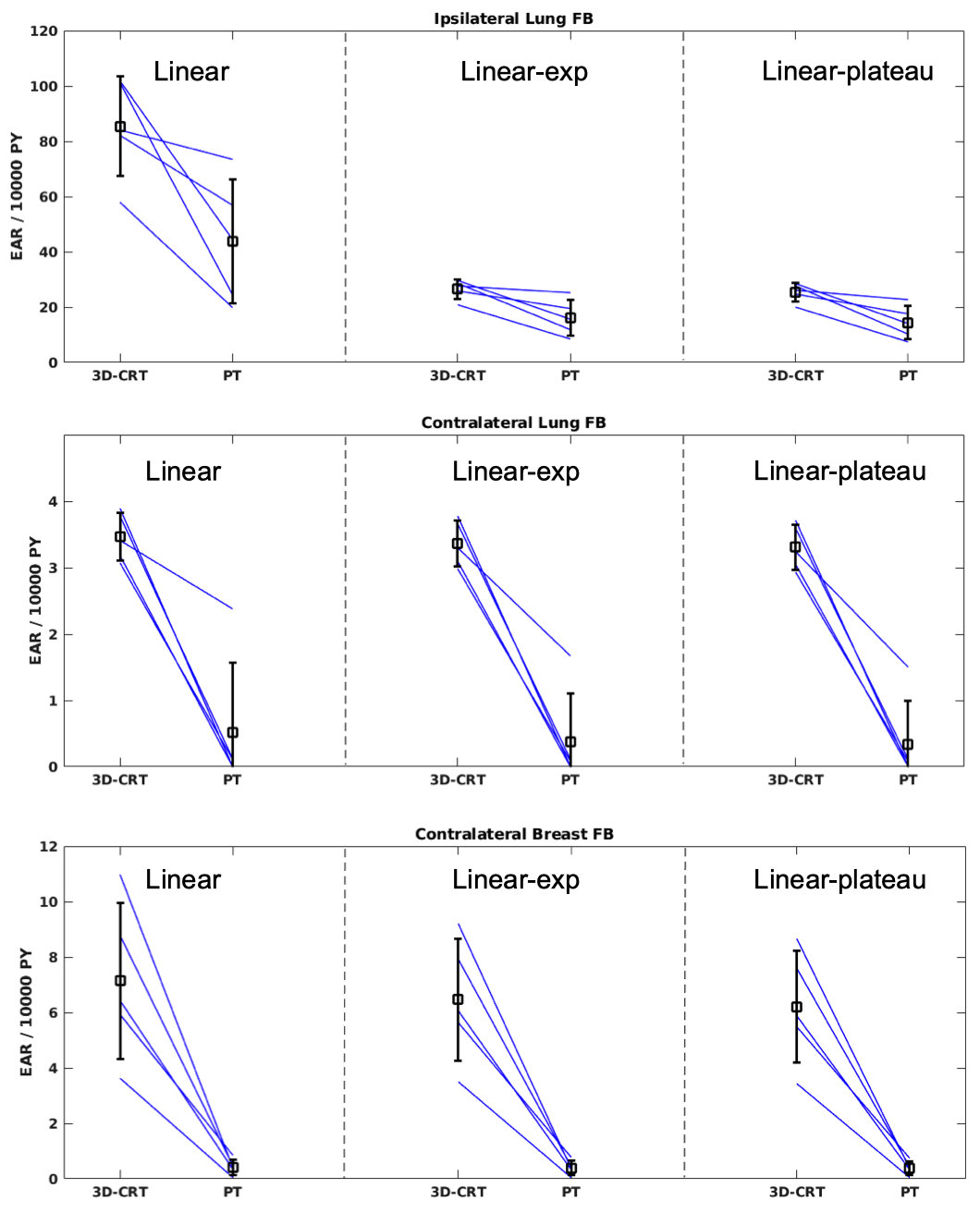

Supplement: Supplementary file 3 — Additional file 3: Figure S3. Average (black squares) and single-patient (blue lines) EAR calculated for the three OARs included in the analysis for VMAT vs PT. Data refer to plans calculated in FB for Group 2. The data displayed refer to patients receiving radiotherapy at the age of 30 years and attaining the age of 70 years. Error bars indicate standard deviation. According to the different absolute values, the Y-axis scale changes for the different OARs. [file 13014_2020_1671_MOESM3_ESM.png]

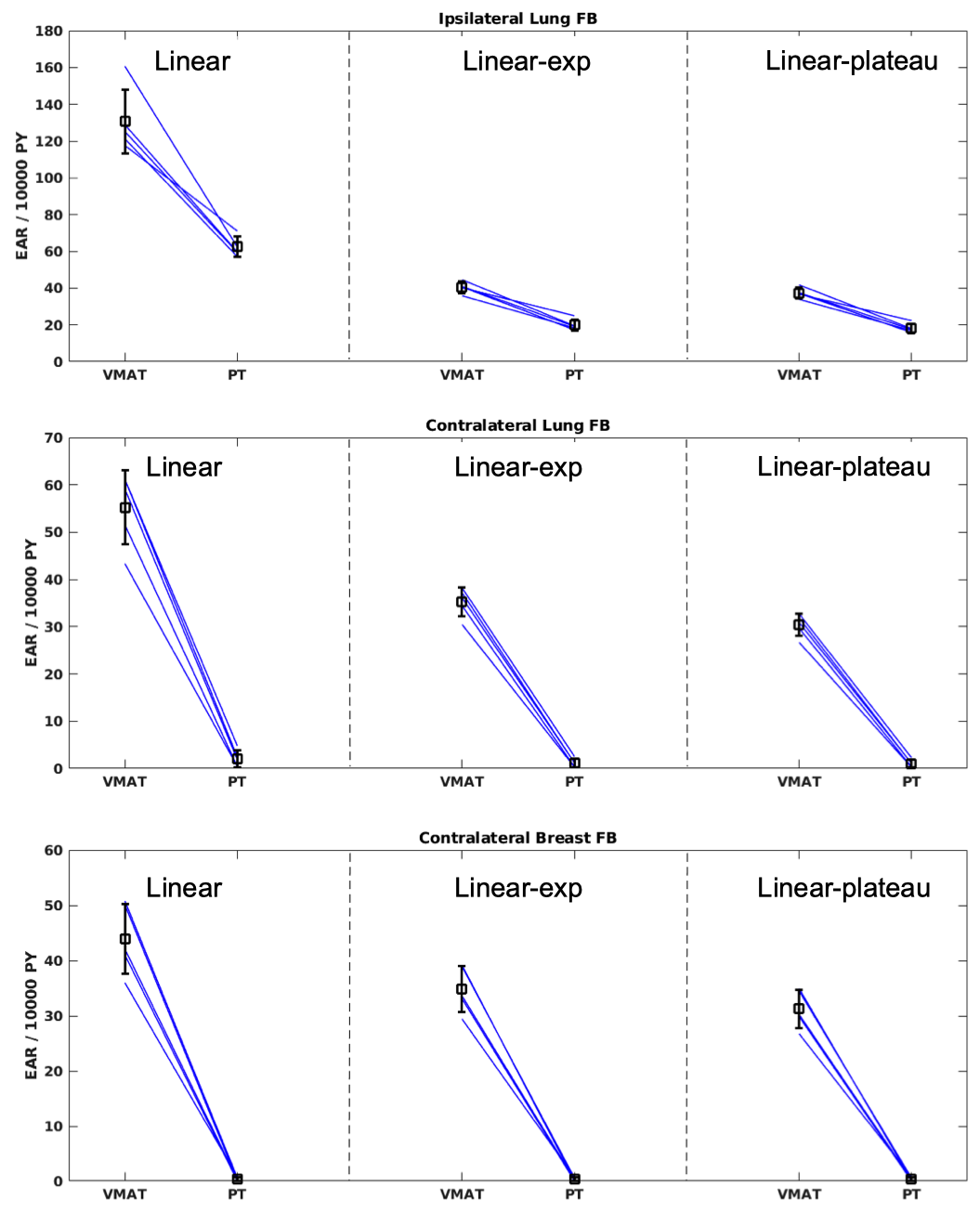

Supplement: Supplementary file 4 — Additional file 4: Figure S4. Average (black squares) and single-patient (blue lines) EAR calculated for the three OARs included in the analysis for VMAT vs PT. Data refer to plans calculated with DIBH for Group 2. The data displayed refer to patients receiving radiotherapy at the age of 30 years and attaining the age of 70 years. Error bars indicate standard deviation. According to the different absolute values, the Y-axis scale changes for the different OARs. [file 13014_2020_1671_MOESM4_ESM.png]
